# A Novel Genetic Variant in *MBD5* Associated with Severe Epilepsy and Intellectual Disability: Potential Implications on Neural Primary Cilia

**DOI:** 10.3390/ijms241612603

**Published:** 2023-08-09

**Authors:** Mariana Martins, Ana Rafaela Oliveira, Solange Martins, José Pedro Vieira, Pedro Perdigão, Ana Rita Fernandes, Luís Pereira de Almeida, Paulo Jorge Palma, Diana Bela Sequeira, João Miguel Marques Santos, Frederico Duque, Guiomar Oliveira, Ana Luísa Cardoso, João Peça, Catarina Morais Seabra

**Affiliations:** 1 Center for Neuroscience and Cell Biology (CNC), University of Coimbra, 3004-504 Coimbra, Portugal; 2 Faculty of Pharmacy, University of Coimbra, 3000-548 Coimbra, Portugal; 3 Institute for Interdisciplinary Research, University of Coimbra, 3030-789 Coimbra, Portugal; 4 Neuropediatrics Unit, Central Lisbon Hospital Center, 1169-045 Lisbon, Portugal; 5 Institute of Endodontics, Faculty of Medicine, University of Coimbra, 3000-075 Coimbra, Portugal; 6 Center for Innovation and Research in Oral Sciences (CIROS), Faculty of Medicine, University of Coimbra, 3000-075 Coimbra, Portugal; 7 University Clinic of Pediatrics, Faculty of Medicine, University of Coimbra, 3000-602 Coimbra, Portugal; 8 Child Developmental Center and Research and Clinical Training Center, Pediatric Hospital, Centro Hospitalar e Universitário de Coimbra (CHUC), 3000-602 Coimbra, Portugal; 9 Department of Life Sciences, University of Coimbra, 3000-456 Coimbra, Portugal

**Keywords:** neurodevelopment, epilepsy, *MBD5*, neural cell models, primary cilia

## Abstract

Disruptions in the *MBD5* gene have been linked with an array of clinical features such as global developmental delay, intellectual disability, autistic-like symptoms, and seizures, through unclear mechanisms. *MBD5* haploinsufficiency has been associated with the disruption of primary cilium-related processes during early cortical development, and this has been reported in many neurodevelopmental disorders. In this study, we describe the clinical history of a 12-year-old child harboring a novel *MBD5* rare variant and presenting psychomotor delay and seizures. To investigate the impact of *MBD5* haploinsufficiency on neural primary cilia, we established a novel patient-derived cell line and used CRISPR-Cas9 technology to create an isogenic control. The patient-derived neural progenitor cells revealed a decrease in the length of primary cilia and in the total number of ciliated cells. This study paves the way to understanding the impact of *MBD5* haploinsufficiency in brain development through its potential impact on neural primary cilia.

## 1. Introduction

Neurodevelopmental disorders (NDDs) are a growing health concern affecting millions of individuals worldwide [1]. NDDs encompass a wide range of conditions, with a common onset during the neurodevelopmental period [2]. Numerous genetic variants are associated with NDDs, encompassing hundreds of different genes [3,4,5]. Disruptions in the *MBD5* gene, such as deletions and duplications, have been linked with NDDs, and the development of an array of clinical features such as global psychomotor delay, intellectual disability, autistic-like symptoms and seizures [6,7]. The main clinical features present in patients with *MBD5* variants and their association between genotype and phenotype are summarized in Table S1. Though this association is known, the exact mechanisms through which perturbed *MBD5* expression leads to neurodevelopmental disorders remain highly underexplored.

The *MBD5* gene includes 5 non-coding exons (1–5) followed by 10 coding exons (6–15), the latter encoding the MBD5 protein [8]. MBD5 is a member of the methyl-CpG-binding domain (MBD) family, which also comprises MBD1-MBD6, SETDB1, SETDB2, BAZ2A, and BAZ2B and MeCP2, the causative locus in Rett syndrome (RTT [MIM 312750]) [9,10]. Given the high expression levels of MBD5 in the brain and the presence of MBD and PWWP domains in its protein structure, it is thought that MBD5 may participate in transcriptional regulation and DNA methylation in the central nervous system (CNS) [8,11,12]. Though the disease mechanisms related to this protein are unclear, a study has found that *MBD5* haploinsufficiency can lead to the disruption of primary cilium-related processes during early cortical development [13]. Furthermore, ciliary defects have been associated with other NDDs such as Rett Syndrome and Fragile X syndrome [14,15].

The primary cilium is a microtubule-based antenna-like organelle that senses and transduces environmental cues during neurodevelopment. Given the large surface area of the primary cilium, this structure can be enriched in a great number and variety of transmembrane receptors, which interact with downstream signaling components [16,17,18]. Through its surface receptors, the primary cilium can detect different hormones, growth factors, morphogens and even neurotransmitters and influence physiological processes [18,19,20,21]. By serving as a “cellular antenna” in the developing central nervous system (CNS), primary cilia can regulate the equilibrium of differentiation and division of neural progenitors, influence the migration of newborn neurons and participate in the proper formation and establishment of neuronal circuits [19,22,23,24,25,26,27].

Likely due to the roles of primary cilia in the development of the CNS, the disruption of this organelle is a common feature in various NDDs [28,29,30]. Given the lack of knowledge regarding *MBD5* variants and the recent findings showing the involvement of primary cilia in NDDs [13], this study aimed to investigate the clinical and cellular alterations present in *MBD5* haploinsufficiency and to further explore the impact of *MBD5* haploinsufficiency on neural primary cilia.

First, we describe the clinical history of a 12-year-old child harboring a novel *MBD5* rare variant, presenting developmental delay, seizures, and Rett-like stereotyped hand movements. Using the patient’s dental stem cells collected from exfoliated teeth, we established and characterized a novel patient-derived induced pluripotent stem cell (iPSC) line and neural progenitor cell (NPC) line. Furthermore, using CRISPR-Cas9 technology, we corrected the *MBD5* variant present in the patient-derived iPSCs, creating an isogenic control. Quantification of cellular and ciliary parameters in NPCs revealed a decrease in the length of neural primary cilia and in the number of ciliated cells.

## 2. Results

### 2.1. Clinical Report of a Patient with Severe Epilepsy and Psychomotor Developmental Delay

The patient is a 12-year-old girl, the second child of healthy, non-consanguineous parents, with no family history of neurologic or neurodevelopmental disorders. The gestation, birth, and neonatal period were uneventful, and the early neurodevelopmental milestones were acquired within normal limits. At the age of 15 months, the patient walked unsupported and, at 18 months, was able to speak a few words, wave goodbye, and point to objects. Since the second half of the first year of life, she exhibited Rett-like stereotyped hand movements. The patient’s acquisition of expressive language at 24 months was below the norm, but she was still making progress, with a vocabulary of 20 words. By the age of four, her quotient of global development on the Griffith Mental Developmental Scale was 77.9 (normal range: 100 ± 15).

**Figure 1:**
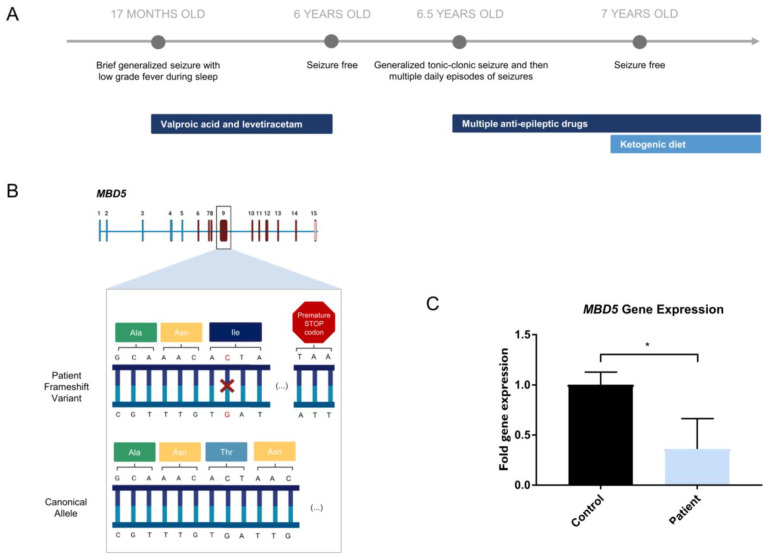
Novel *MBD5* variant harbored by a patient with severe epilepsy. (**A**) Clinical history of patient with *MBD5* variant presenting with severe epilepsy; (**B**) Representation of the heterozygous single nucleotide deletion (highlighted in red) in the *MBD5* gene (NM_018328.4), found in the patient, expected to create a premature STOP codon in exon 9 (created with BioRender.com, accessed on 5 June 2022); (**C**) Patient-derived NPCs reveal lower expression of *MBD5* in patient-derived NPCs, in transcripts that include exons downstream from the variant (Student’s *t*-test; Data are presented as mean ± s.d. Statistical significance: * *p* < 0.05).

The patient’s clinical course of epilepsy started when she was 17 months old when during sleep, she had a brief generalized seizure with low-grade fever. Brain MRI showed no structural abnormalities, but an electroencephalogram (EEG) revealed bilateral frontal spikes and slightly slow background activity. Treatment with valproic acid was started, and later, levetiracetam was added due to continuing seizures. The patient was seizure-free when she was six years old, so treatment with valproic acid and levetiracetam was gradually discontinued. An EEG at this time still displayed infrequent frontal epileptiform activity. After six months with no treatment, the patient had a brief episode of “staring” followed by a generalized tonic–clonic seizure. Subsequently, she had multiple daily episodes of both types of seizures, which were refractory to multiple antiepileptic drugs, namely valproic acid, levetiracetam, lacosamide, ethosuximide, perampanel, and prednisolone. EEGs revealed generalized spike-wave and polyspike-wave activity, strongly activated during sleep. After starting a ketogenic diet along with her medication, the patient subsequently had a prompt and complete response, becoming seizure-free again (Figure 1A).

When the patient was seven years old, next-generation sequencing (NGS), sequencing of 228 genes related to epileptic encephalopathies was performed, identifying a heterozygous mutation in the *MBD5* gene (Figure 1B; Table S6). At age 12, the patient still displays Rett-like stereotyped hand movements without hand apraxia. The patient is in regular school with curriculum adaptation. Though she is unable to read or write, she can recognize letters, numbers, and a few words. Physically, growth (weight and height) is at the 75th percentile, and head circumference is at the 75–90th percentile. The mouth is wide, and teeth are widely spaced and abnormal in shape. There is a fifth finger clinodactyly, lower limb reflexes are brisk and distal tone is slightly increased. Plantar responses are flexor, and neurologic examination is otherwise normal.

### 2.2. Novel MBD5 Genetic Variant Shows Haploinsufficiency

Though the patient in the study presented a phenotype partially consistent with Rett syndrome, previous tests ordered by Central Lisbon Hospital Center (Portugal) showed that there were no alterations in the *MECP2* gene, the responsible locus for this condition. Thus, the patient was indicated for further genetic testing using an NGS panel to investigate a possible genetic cause for the symptoms (Table S6). The results of the NGS panel indicated that the patient carried a heterozygous variant in the *MBD5* gene (NM_018328.4)—c.2297del (p.(Thr766Ilefs*18)) in exon 9, consisting of a single nucleotide deletion in the position 2297 of the open reading frame, which leads to a premature STOP codon (Figure 1B). According to the prediction tool PolyPhen-2, the *MBD5* variant harbored by the patient is possibly damaging with a score of 0.801, which is confirmed by the clinical phenotype presented. Gene expression assessment downstream of the deletion site in patient-derived cells shows a decrease in *MBD5* expression (Student’s t-test; statistical significance: * *p* < 0.05), consistent with haploinsufficiency (Figure 1C). We have also assessed gene expression of exons 5–6 upstream of the deletion site (Figure S2B). This showed a trend toward a decrease in *MBD5* expression, although not reaching statistical significance. This indicates that while shorter mRNA transcripts may be maintained, the mutated allele will produce a premature termination codon of the longer canonical transcript, which will lead to its degradation by the nonsense-mediated mRNA decay pathway [31,32].

### 2.3. Establishment of an iPSC Cell Line from Patient-Derived Dental Stem Cells

**Figure 2:**
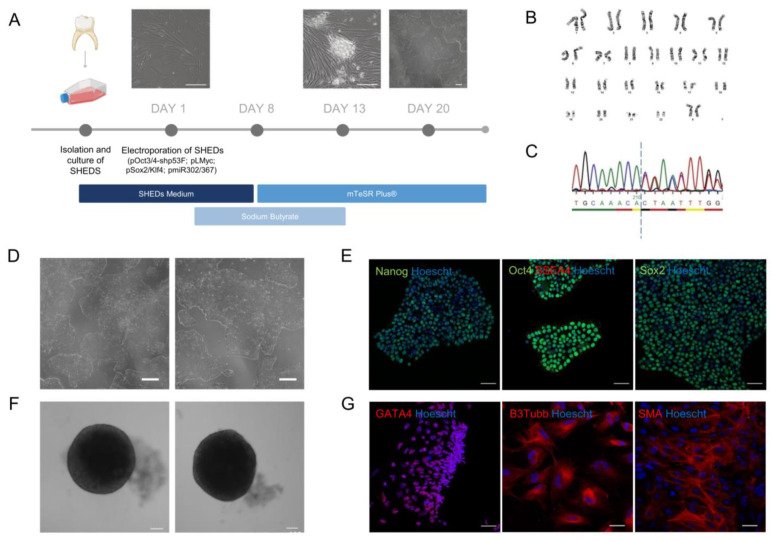
Establishment of a patient-derived iPSC line. (**A**) Timeline of SHEDs reprogramming (scale bar: 200 µm); (**B**) A normal karyotype (46, XX) was identified in the patient-derived iPSCs; (**C**) Sanger Sequencing of the of the loci in exon 9 in patient-derived iPSCs reveals the variant harbored by the patient, creating a frameshift after the dashed line; (**D**) Patient-derived iPSCs presenting typical pluripotent morphology following reprogramming (scale bar: 200 µm); (**E**) Patient-derived iPSCs reveal expression of pluripotency markers OCT4, NANOG, SSEA4 and SOX2 (objective: 20×, scale bar: 50 µm); (**F**) EBs generated from patient-derived iPSCs display a spherical and well-defined morphology (scale bar: 100 µm); (**G**) Patient-derived iPSCs are able to differentiate into the three embryonic germ layers and present expression of trilineage markers GATA4, B3Tubb and SMA (objective: 20×, scale bar: 50 µm).

With the goal of studying the primary cilia consequences of the *MBD5* variant in human neural cells, we generated iPSCs from patient-derived stem cells from human exfoliated deciduous teeth (SHEDs) that can be used for downstream neural studies. For this purpose, a deciduous tooth was collected from the patient in the study, and the SHEDs were cultured until a stable population was obtained. The SHEDs were then reprogrammed according to Howden et al. [33] (Figure 2A) via electroporation using episomal-based reprogramming constructs. A single clone was chosen and expanded until cells demonstrated an appearance typical of iPSCs, with a high nucleus-to-cytoplasm ratio organized in round-shaped well-defined colonies (Figure 2D).

To ensure that no chromosomal aberrations occurred in the reprogramming of newly generated iPSCs, we performed karyotyping of selected clones before downstream characterization [34,35]. Using conventional cytogenetics, a normal karyotype was ascertained in the iPSCs, which presented 22 pairs of autosomes and two sex chromosomes (XX) (Figure 2B). Moreover, Sanger Sequencing confirmed that the iPSCs preserved the *MBD5* deletion (NM_018328.4)—c.2297del (p.(Thr766Ilefs*18)) in exon nine harbored by the patient (Figure 2C).

To validate the pluripotency of the newly generated iPSCs, an immunofluorescence staining was performed for the standard pluripotency markers SSEA4, OCT4, and SOX2, where all stained positively for these markers (Figure 2E). Additionally, these cells were able to form embryoid bodies (EBs) (Figure 2F) and differentiated into the three embryonic germ layers, as confirmed by immunofluorescence staining and confocal microscopy. Cells expressed markers specific to each corresponding embryonic layer, including β-tubulin III (TUBB3), a marker of ectoderm; smooth muscle actin (SMA), indicative of mesoderm; and GATA4, an endoderm marker (Figure 2G).

### 2.4. CRISPR-Cas9 HDR-Mediated Correction the MBD5 Variant in the Patient-Derived iPSCs

To properly assess the ciliary phenotypes that result specifically from the *MBD5* variant, we created an isogenic control to account for any differences in the genetic background that could influence this phenotype. For this purpose, homology-directed repair (HDR) was performed to correct the *MBD5* deletion. The patient-derived iPSC line harbors a heterozygous cytidine deletion (c.2297delC) within the exon nine coding region of *MBD5* that leads to disruption of the coding frame and loss of MBD5 expression. Two guide RNAs (gRNAs) were designed—MBD5 g9.1 and MBD5 g9.2—to target the c.2297delC region in conjugation with Cas9 endonuclease as ribonucleoprotein (RNP) complexes (Figure S2A). Two single-stranded deoxyoligonucleotide (ssODN) templates—ssODN9.1 and ssODN9.2—with homologous regions to the *MBD5* exon nine were designed to be co-delivered respectively with MBD5 g9.1 or MBD5 g9.2-conjugated RNP for targeted knock-in of the missing cytidine. Additional silent mutations were inserted in the ssODN templates (highlighted in red) to block Cas9 recognition and cleavage following HDR. Targeted *MBD5* indels resulting from non-homologous end joining (NHEJ) repair or precise HDR-mediated c.2297delC correction was estimated by Inference of CRISPR Edits software (ICE Version 3.0). *MBD5* edited clones are distributed according to ssODN knock-in (none, heterozygous or homozygous). *MBD5* gene correction with the restoration of the coding frame only occurred in homozygous ssODN knock-in clones (Figure S1B–D). The corrected iPSC line showed pluripotency markers OCT4 and SSEA4 (Figure S2A).

### 2.5. NPCs Reveal Neural Identity Markers

**Figure 3:**
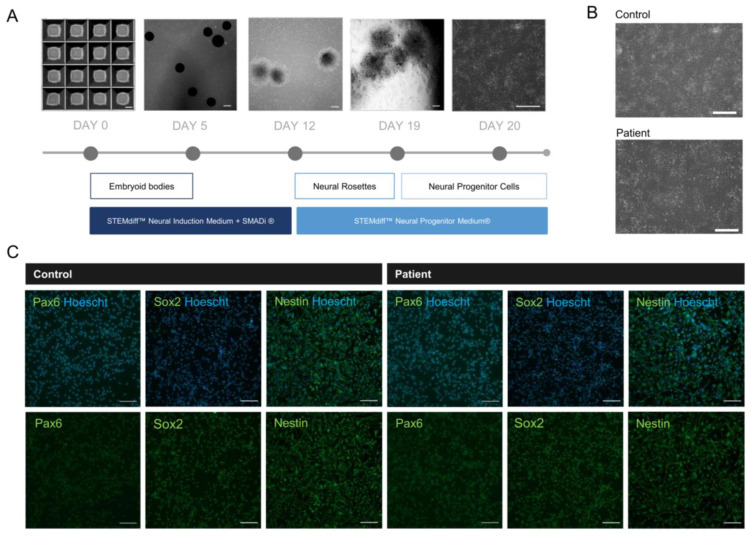
Development of 2D neural progenitor cells from iPSCs reveals successful differentiation. (**A**) Timeline of neural progenitor cell differentiation (scale bar: 200 µm); (**B**) Patient-derived NPCs presenting typical neural progenitor morphology following differentiation (scale bar: 200 µm); (**C**) NPCs reveal expression of neural progenitor markers Pax6, Sox2 and Nestin (objective: 20×, scale bar: 100 µm).

As a cellular model of neurodevelopment, NPCs derived from patient iPSCs allow for the study of the pathological features of *MBD5* haploinsufficiency and the role of primary cilia in this condition [36]. The patient-derived and isogenic control NPCs were generated from iPSCs using the STEMdiff™ SMADi Neural Induction Kit (STEMCELL Technologies) (Figure 3A,B). The identity of the NPCs was confirmed by immunofluorescence, revealing the presence of neural identity markers Pax6, Sox2, and Nestin (Figure 3C) and the absence of pluripotency marker OCT4 (Figure S2B). Two MBD5 antibodies were tested, but the obtained staining was unspecific (Figure S2C).

### 2.6. Evaluation of Primary Cilia Markers and Length in NPCs Shows Alterations in Arl13b+ Cilia Length in Patient Cells

**Figure 4:**
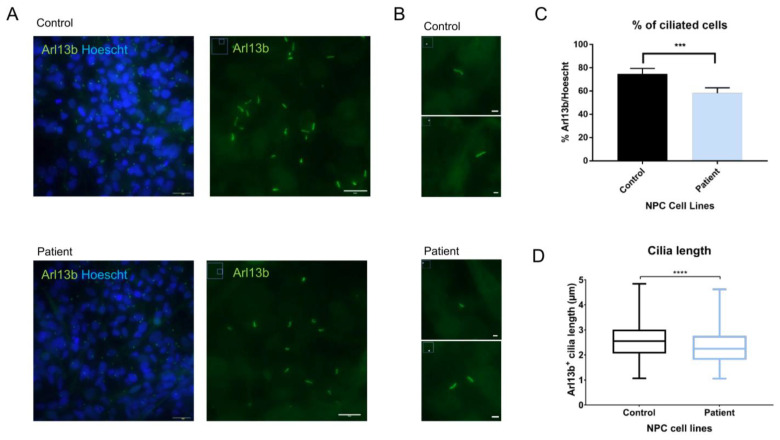
Measurement of Arl13b+ primary cilia show alterations in cilia length and number in patient-derived NPCs. (**A**) NPCs reveal expression of primary cilia marker Arl13b (objective: 63×, scale bar: 24 µm on Arl13b/Hoescht; 10 µm on Arl13b); (**B**) Arl13b+ cilia with 200% zoom observed on ImageJ (scale bar: 2 µm); (**C**) Primary cilia counting using ImageJ reveals a lower number of Arl13b+ primary cilia in patient-derived NPCs (Student’s *t*-test; Statistical significance: *** *p* = 0.0005); (**D**) Measurement of primary cilia apparent length using ImageJ reveals shorter primary cilia in patient-derived NPCs when compared to isogenic controls (Student’s *t*-test; Statistical significance: **** *p* < 0.0001).

To understand the potential implications of *MBD5* haploinsufficiency on primary cilia, we studied the ciliary length and total cilia count, as these have been identified in other neurodevelopmental disorders [14,15,37]. Using immunofluorescence, we stained control and patient-derived NPCs for cilia-enriched small GTPase Arl13b and compared primary cilia length and number. Our results indicate that Arl13b is present in the NPCs, delineating the primary cilia (Figure 4A,B). Importantly, compared to isogenic controls, patient-derived NPCs showed a decrease in cilia length (Student’s *t*-test; Statistical significance: **** *p* < 0.0001; Figure 4C) and in the number of ciliated cells (Student’s *t*-test; Statistical significance: *** *p* = 0.0005; Figure 4D), a phenotype observed in NDDs such as Rett Syndrome and Fragile-X syndrome [14,15].

## 3. Discussion

In modern societies, neurodevelopmental disorders (NDDs) have become increasingly prevalent conditions. Though NDDs affect millions of individuals, their complex and heterogenous nature makes it difficult to uncover the mechanisms behind these disorders. Even so, hundreds of genes and biological pathways have been related to NDDs. *MBD5* variants are often associated with neurological symptoms, including psychomotor developmental delays and seizures. In this study, we describe a rare genetic variant in *MBD5* (NM_018328.4)—c.2297del (p.(Thr766Ilefs*18)), the only reported case in Portugal, from a 12-year-old female presenting Rett-like phenotype, developmental delay, and drug-resistant seizures. Despite a variety of drugs prescribed to treat the patient’s epilepsy, only a ketogenic diet seemed to resolve the seizures. This has been effective in children with drug-resistant epilepsy, and it is especially beneficial in conditions such as Rett Syndrome, Angelman syndrome, and Tuberous Sclerosis Complex [38,39,40,41]. The anti-seizure and neuroprotective effects of the ketogenic diet are thought to happen due to several synergistic mechanisms, including alterations in neurotransmitter levels, improvement of mitochondrial biogenesis and function, and changes in gut microbiota [42,43,44,45,46].

Though the ketogenic diet was a successful therapeutic strategy to treat the patient’s epilepsy, the neurodevelopmental symptoms remain without treatment. In an effort to understand the cellular impact of *MBD5* haploinsufficiency and thus reach potential therapeutic targets from the patient’s dental cells, we generated and characterized a novel iPSC line that maintains the patient’s deletion. To properly validate the pluripotency of the newly generated iPSCs, quality control assays were employed, following the currently accepted tests for iPSC characterization [47]. Furthermore, using CRISPR-Cas9 technology, we corrected the *MBD5* variant creating an isogenic control that allowed us to study the individual contribution of the novel variant in NDDs. Future studies should include additional control cell lines to ensure that no off-target effects from gene editing are contributing to the phenotypes observed.

The differentiation of iPSC into NPCs allowed us to explore the potential impact of *MBD5* on neural ciliary function. We observed lower expression of *MBD5* in patient-derived NPCs in transcripts that include exons downstream from the variant. These results reveal that the mRNA containing a premature termination codon could have been degraded via the nonsense-mediated mRNA decay pathway [31]. Regarding primary cilia alterations, we observed lower cilia count and shorter length in Arl13b stained-cilia of the patient-derived NPCs when compared to CRISPR-Cas9-edited cells. This phenotype has been observed in other NDDs, such as the ones caused by variants in *mTOR*, *Fmr1*, and *MecP2* [14,15,37]. When it comes to NDDs caused by *mTOR* variants, mouse models and patient neurons display primary cilia with significant reductions in number and length. In the case of *MECP2* deficiency, ciliogenesis was disrupted in mouse brain and human patient fibroblasts, which displayed a lower percentage of ciliated cells and lower cilia length [14]. Furthermore, in Fragile X Syndrome, *Fmr1* knockout mice presented primary ciliary deficits in the dentate gyrus of the hippocampus, also presenting lower cilia count and ciliary length [15].

Though these studies have presented a clear link between ciliary alterations and the mechanisms of NDDs, none have utilized patient-derived NPCs, which are key players in the early formation of the CNS that use their primary cilia to control the equilibrium between proliferation and differentiation to other cell types [22,48,49,50]. Moreover, an *MBD5* haploinsufficiency NPC model was previously reported but did not explore alterations in primary cilia [51].

The mechanism underlying the link between *MBD5* and primary cilia requires further understanding. In an analogous manner to what is observed in Rett syndrome, it is possible that the ciliary defects observed can be induced by the transcriptional functions of *MBD5* [14]. Moreover, a recent study showed that *MBD5* overexpression inhibited *Stat1* transcription, which resulted in increased expression of N-methyl-D-aspartate receptors (NMDARs), which led to aggravation of epileptic behavior in mice [52]. Another study showed that NMDAR antagonists lead to the shortening of primary cilia [53]. Altogether, this could suggest that haploinsufficiency of *MBD5* could lead to decreased NMDAR expression, which would have similar effects to an antagonist and thus lead to primary cilia shortening. This should be further explored using high-resolution microscopy and advanced realistic models of human brain development, such as brain organoids.

Altogether, this study contributes to describing a novel rare variant in *MBD5* and to pave the way to understanding the impact of *MBD5* haploinsufficiency in brain development through its impact on neural primary cilia.

## 4. Materials and Methods

### 4.1. Culture and Cryopreservation of SHEDs

A deciduous tooth from an individual with a heterozygous *MBD5* deletion (12 years of age, *MBD5*^+/−^) was collected following approval by the Ethics Committees from both the Coimbra University Hospital (#CHUC-049-18) and the Faculty of Medicine, University of Coimbra (#121-CE-2017) and in agreement with the principles for medical research in the Declaration of Helsinki. Informed consent was obtained from the legal guardians, and data were treated confidentially according to the General Data Protection Regulation (GDPR), Regulation (EU) 2016/679 of the European Parliament and of the Council of 27 April 2016. Stem cells from human exfoliated deciduous teeth (SHEDs) were collected from the teeth pulp using a sterile needle, and the tissue was cut using a scalpel blade. The sample was then incubated at 37 °C in digestion medium composed of DPBS (Biowest, Nuaillé, France), Penicillin/Streptomycin (Pen/Strep) (Gibco Chemicals—Thermo Fisher Scientific, Waltham, MA USA), collagenase type I (Gibco Chemicals—Thermo Fisher Scientific, Waltham, MA USA), and dispase II (Gibco Chemicals—Thermo Fisher Scientific, Waltham, MA USA). After incubation, the digested fragments were passed through a 70 µm strainer and centrifuged at 300 g for 3 min. The cell pellet was then transferred to a T25 flask with 5 mL of SHEDs media, composed of KO-DMEM (Gibco Chemicals—Thermo Fisher Scientific, Waltham, MA USA) supplemented with 20% fetal bovine serum heat-inactivated (FBS-HI) (Biowest, Nuaillé, France), 1% GlutaMax (Gibco Chemicals—Thermo Fisher Scientific, Waltham, MA USA), 1% β-mercaptoethanol (Merck, Rahway, NJ, USA) and 1% Pen/Strep (Gibco Chemicals—Thermo Fisher Scientific, Waltham, MA USA). The SHEDs were cultured in T175 flasks and incubated at 37 °C in a humidified atmosphere with 5% CO_2_. Media was replaced every 3 days, and cells were passaged when 70% confluency was reached, using dissociation media containing 8 g/L of NaCl, 1.16 g/L of Na_2_HPO_4_ (anhydrous), 0.2 g/L of KH_2_PO_4_ (anhydrous), 0.2 g/L of KCl, 0.16 g/L of EDTA and 2% trypsin.

### 4.2. Reprogramming Patient-Derived SHEDs into iPSCs

SHEDs were reprogrammed according to Howden et al. (2015) and adjusted for an initial amount of 2.5 × 10^6^ cells [33]. After electroporation, SHEDs were plated on a single 10 cm tissue culture dish coated with gelatin 0.1% in DPBS and maintained in SHEDs media. The following day, media was switched to SHEDs media containing 0.5 mM sodium butyrate (SB) (STEMCELL Technologies, Saint Égrève, France) and replaced daily until day 6. On day 7, the cells were dissociated with TrypLE, plated on a 6-multiwell plate previously coated with Corning Matrigel^®^ hESC-qualified matrix (Corning, Inc., Corning, NY, USA), and maintained in SHEDs media supplemented with 0.5 mM SB. From day 8 onward, the cells were cultured in mTeSR™ Plus medium (STEMCELL Technologies, Saint Égrève, France) with 0.5 mM SB431542, changed every two days. SB431542 was removed from the media on day 13, and the media was changed every two days. iPSC colonies emerged around day 15 and were manually scraped with a 10 µm tip and collected, maintained, and expanded on Corning Matrigel^®^ hESC-qualified matrix-coated (Corning, Inc., Corning, NY, USA) 6-multiwell plates in mTeSR™Plus medium (STEMCELL Technologies, Saint Égrève, France) supplemented with 1% of Pen/Strep (Gibco Chemicals—Thermo Fisher Scientific, Waltham, MA USA), with media changes every two days and passages every four to five days with ReleSR passaging reagent (STEMCELL Technologies, Saint Égrève, France).

### 4.3. Immunostaining of iPSCs

iPSCs were plated on μ-slide 8-well ibiTreat chamber slides (Ibidi, Gräfelfing, Germany), coated with Matrigel^®^ (Corning, Inc., Corning, NY, USA), and maintained with mTeSR™Plus medium (STEMCELL Technologies, Saint Égrève, France). After reaching around 70% confluency, cells were fixed with 4% paraformaldehyde (PFA), followed by primary antibody incubation with human protein-reactive anti-SSEA4 (1:66, Abcam, Cambridge, UK, ab16287), anti-OCT4 (1:1000, Abcam, Cambridge, UK, ab19857), anti-SOX2 (1:1000, Abcam, Cambridge, UK, ab97959) and anti-Nanog (1:1000, Abcam, Cambridge, UK, ab21624). Nuclei were stained with Hoechst 33342 (1 μg/mL), and cells were then observed under a Zeiss LSM 710 confocal microscope (Zeiss, Baden-Württemberg, Germany) at 20× magnification.

### 4.4. Trilineage Differentiation of iPSCs

The trilineage differentiation experiment was carried out as described by Varga et al. [54] to assess the iPSCs’ ability to differentiate into EBs and into the three germ layers. Embryoid bodies (EBs) were generated using the STEMdiff™ Cerebral Organoid Kit (STEMCELL Technologies, Saint Égrève, France), according to the manufacturer’s instructions. On day 5, the EBs displayed a spherical and well-limited appearance, typical of EBs formed from iPSCs. The EBs were plated on μ-slide 8-well ibiTreat chamber slides (Ibidi, Gräfelfing, Germany), coated with Matrigel^®^ (Corning, Inc., Corning, NY, USA), and maintained in 3 germ layer differentiation medium: DMEM/F12 (Gibco Chemicals - Thermo Fisher Scientific, Waltham, MA USA) supplemented with 20% FBS (Biowest), 1% MEM Non-Essential Amino Acid Solution (Gibco Chemicals—Thermo Fisher Scientific, Waltham, MA USA), 0.1 mM β-mercaptoethanol (Merck, Rahway, NJ, USA) and 1% Pen/Strep (Gibco Chemicals—Thermo Fisher Scientific, Waltham, MA USA). The medium was changed every other day until day 14 of differentiation. On day 14, cells were fixed with 4% PFA. The presence of endoderm, mesoderm, and ectoderm was confirmed using, respectively, anti-GATA4 (1:500 dilution, Santa Cruz Biotechnology, Dallas, TX, USA, sc-25310), anti-SMA (1:200, Agilent Dako, Santa Clara, CA, USA, M0851), and β3Tubulin (1: 200, Merck Millipore, Rahway, NJ, USA, #MAB1637) (Figure 4). To confirm the presence of the pluripotency markers, the samples were observed under a Zeiss LSM 710 confocal microscope (Zeiss, Baden-Württemberg, Germany), with a magnification of 20×.

### 4.5. Sanger Sequencing

Following the clinical finding of a rare variant detected in the *MDB5* gene via NGS, DNA was extracted from the patient-derived iPSC pellets using the NZY Tissue gDNA Isolation kit (NZYTech, Lisbon, Portugal), following the manufacturer’s instructions. The DNA sample to be sequenced was combined in a microtube with primers for the region of interest, previously designed using Primer-BLAST (Table S2). The samples were then sent to GATC Biotech (Constance, Germany) for Sanger Sequencing.

### 4.6. Karyotyping

The karyotype analysis was performed by a certificated Genetic Laboratory (Centro de Medicina Laboratorial Germano de Sousa, Portugal) using standard G-banding techniques. Briefly, iPSCs were cultured in a Corning^®^ Matrigel^®^-coated T25 flask and treated with 10 µg/mL Colcemid (0.05 µg/mL final) for up to 1 h, followed by dissociation with ACCUTASE^®^. The iPSCs were then centrifuged and the resulting pellet was re-suspended in pre-warmed 0.075 M KCl hypotonic solution and incubated for 5 min at 37 °C. After centrifugations, the cells were re-suspended in Carnoy’s fixative (3:1 ratio of methanol: glacial acetic acid). Metaphase spreads were prepared on glass microscope slides and GTG-banded by brief exposure to trypsin and stained with Leishman. For the patient-derived iPSCs, 50 metaphases were analyzed, and the karyotype was established in accordance with the International System for Human Cytogenetic Nomenclature (ISCN) 2020.

### 4.7. Correction of MBD5 c.2297delC in iPSCs via CRISPR/Cas9

Gene-corrected isogenic iPSCs were generated via homology-directed repair (HDR) of *MBD5* c.2297delC by co-delivery of CRISPR/Cas9 ribonucleoprotein (RNP) targeting *MBD5* and single-stranded oligodeoxynucleotide (ssODN) donor templates. CRISPR/Cas9 guide RNAs (gRNAs) and ssODN templates were designed using CRISPR design software Benchling (www.benchling.com, Benchling for Academics, accessed since 1 January 2022). *MBD5* gRNAs were designed to target the exon 9 close to the c.2297delC mutation and ordered for downstream assembly with Cas9 endonuclease. Donor ssODN were ordered as Ultramer oligos. All reagents were purchased from Integrated DNA Technologies (IDT). *MBD5* gRNAs target sequences and ssODN sequences are presented in Table S3. The patient-derived iPSC line was nucleofected with *MBD5*-targeting RNP and ssODN donors using a Lonza 4D-nucleofector with X-unit (Lonza, Basel, Switzerland) in P3 Primary cell nucleofector solution and using electroporation program CA-137. Cells were seeded into Matrigel-coated 24-well plates in the presence or absence of 30 µM HDR enhancer (HDRe, IDT, Leuven, Belgium). Five days post-nucleofection, genomic DNA was extracted, and HDR efficacy was estimated via analysis of Sanger sequences by Inference of CRISPR edits software (ICE Version 3.0). Gene-edited single cell-derived clones were then isolated and validated by Sanger sequencing. Primers used for genotyping *MBD5* c.2297delC correction are presented in Table S4.

### 4.8. Neural Progenitor Cell Differentiation

Neural progenitor cells were differentiated from iPSCs via the embryoid body (EB) protocol using STEMdiff™ SMADi Neural Induction Kit (STEMCELL Technologies, Saint Égrève, France), according to the manufacturer’s instructions. On day 19, when NPCs formed a confluent monolayer around the rosette clusters, the cells were passaged using ACCUTASE™ (STEMCELL Technologies, Saint Égrève, France), centrifuged, and counted using Trypan Blue. Around 1.25 × 10^6^ cells were plated per well of 6-well plate coated with Corning Matrigel^®^ and maintained in STEMdiff™ Neural Progenitor Medium (STEMCELL Technologies, Saint Égrève, France). From this day onward, daily medium changes were performed, with passages made once a week, in which cells were plated at a density of 1.25 × 10^5^ cells/cm^2^. To maintain a stock of NPCs for future experiments, each time a passage was made, 4 × 10^6^ cells were frozen per cryovial (Corning, Inc., Corning, NY, USA) in STEMdiff™ Neural Progenitor Freezing Medium (STEMCELL Technologies, Saint Égrève, France) using a slow rate-controlled cooling container in an ultra-low temperature freezer at −80 °C.

### 4.9. Immunostaining of NPCs

NPCs were plated on μ-slide 8-well ibiTreat chamber slides (Ibidi, Gräfelfing, Germany), coated with Matrigel^®^, at a density of 1.25 × 10^5^ cells/cm^2^ and maintained with STEMdiff™ Neural Progenitor Medium (STEMCELL Technologies, Saint Égrève, France). Cells were fixed with 4% paraformaldehyde (PFA), followed by primary antibody incubation with human protein-reactive anti-Pax6 (1:100, Invitrogen, Thermo Fisher Scientific, Waltham, MA USA, 42-6600), anti-SOX2 (1:1000, Abcam, Cambridge, UK, ab97959), anti-Nestin (1:500, Millipore, ABD69), anti-NeuN (1:100, Merck Millipore, Rahway, NJ, USA, #MAB377), and anti-Arl13b (1:200, Proteintech, Rosemont, IL, USA, 17711-1-AP), anti-MBD5 (1:500, Proteintech, 15961-1-AP) and anti-MBD5 (1:200, Merck Sigma-Aldrich, Rahway, NJ, USA, WH0055777M1) properly diluted in a 3% BSA in DPBS solution, overnight at 4 °C. Nuclei were stained with Hoechst 33342 (1 μg/mL) and cells were then observed under a Zeiss LSM 710 confocal microscope (Zeiss, Baden-Württemberg, Germany) at 20× magnification.

### 4.10. Quantitative Real-Time Polymerase Chain Reaction (qRT-PCR)

RNA was isolated from patient-derived and control NPCs from three independent passages using the NucleoSpin^®^ RNA kit (Macherey-Nagel, Nordrhein-Westfalen, Germany), according to the manufacturer’s instructions. The quality and concentration of the RNA were determined using a NanoDrop spectrophotometer (Thermo Fisher Scientific, Waltham, MA USA). First-strand cDNA synthesis was then carried out with the NZY First-Strand cDNA Synthesis Kit (NZYTech, Lisbon, Portugal), according to the manufacturer’s instructions. Quantitative real-time polymerase chain reaction (qRT-PCR) was performed in a CFX96 Touch Real-Time PCR Detection System (Bio-Rad) with the SsoAdvanced Universal SYBR Green Supermix (Bio-Rad, Hercules, CA, USA), following the manufacturer’s protocol and using two technical replicates. Primers were designed using Ensembl (Release 106) and Primer-Blast software (Table S5). Samples were normalized to the housekeeping gene *GAPDH.* The acquired data were analyzed with the QuantStudio^®^ Software (Thermo Fisher Scientific, Waltham, MA USA, Design and Analysis Version 2.0), and the relative differences in transcript levels were assessed using the ΔΔCt method.

### 4.11. Primary Cilia Count and Length Analysis

For primary cilia counting and length measurement, images were acquired from the Arl13b staining of CRISPR-Cas9-edited and patient-derived NPCs after 5 passages post-induction, using a Zeiss Observer microscope (Zeiss, Baden-Württemberg, Germany) through a 63× oil-immersion objective. The cilia-enriched small GTPase Arl13b was chosen as a neural progenitor primary cilia marker due to its presence in neural tube patterning and the formation of the polarized radial glial scaffold [25,55]. With ImageJ software (Version 1.53t), eight maximum-intensity projection images were obtained, and the total number and apparent length of Arl13b+ primary cilia were assessed using the multi-point tool and the line selection tool, respectively. Regarding ciliary length, a total of 270 cilia were counted per patient and control lines. Isolated spots that were brighter than the Arl13b staining were excluded from the counting and measurement analysis. Cilia that presented gaps in their apparent length were also excluded from the measurement analysis. The graphical and statistical analysis was performed using GraphPad Prism 7.0, where a Student’s *t*-test was employed to compare the means between two groups (GraphPad Software, Version 7.0) [56].

## Data Availability

Data are available upon request to corresponding authors.

## Supplementary Materials

The supporting information can be downloaded at: https://www.mdpi.com/article/10.3390/ijms241612603/s1, References [57,58,59,60,61,62,63,64,65,66,67,68,69,70,71,72,73,74,75,76,77,78,79,80,81,82,83,84,85,86,87,88,89] are cited in the supplementary materials (Table S1).

## References

[1] Francés L., Quintero J., Fernández A., Ruiz A., Caules J., Fillon G., Hervás A., Soler C.V. (2022). Current state of knowledge on the prevalence of neurodevelopmental disorders in childhood according to the DSM-5: A systematic review in accordance with the PRISMA criteria. Child Adolesc. Psychiatry Ment. Health.

[2] American Psychiatric Association (2013). Diagnostic and Statistical Manual of Mental Disorders.

[3] Cardoso A.R., Lopes-Marques M., Silva R.M., Serrano C., Amorim A., Prata M.J., Azevedo L. (2019). Essential genetic findings in neurodevelopmental disorders. Hum. Genom..

[4] De Rubeis S., He X., Goldberg A.P., Poultney C.S., Samocha K., Cicek A.E., Kou Y., Liu L., Fromer M., Walker S. (2014). Synaptic, transcriptional and chromatin genes disrupted in autism. Nature.

[5] Peça J., Feng G. (2012). Cellular and synaptic network defects in autism. Curr. Opin. Neurobiol..

[6] Talkowski M.E., Mullegama S.V., Rosenfeld J.A., van Bon B.W.M.M., Shen Y., Repnikova E.A., Gastier-Foster J., Thrush D.L., Kathiresan S., Ruderfer D.M. (2011). Assessment of 2q23.1 microdeletion syndrome implicates MBD5 as a single causal locus of intellectual disability, epilepsy, and autism spectrum disorder. Am. J. Hum. Genet..

[7] Mullegama S.V., Elsea S.H. (2016). Clinical and Molecular Aspects of MBD5-Associated Neurodevelopmental Disorder (MAND). Eur. J. Hum. Genet..

[8] Laget S., Joulie M., Le Masson F., Sasai N., Christians E., Pradhan S., Roberts R.J., Defossez P.-A.A. (2010). The human proteins MBD5 and MBD6 associate with heterochromatin but they do not bind methylated DNA. PLoS ONE.

[9] Klose R.J., Bird A.P. (2006). Genomic DNA methylation: The mark and its mediators. Trends Biochem. Sci..

[10] Roloff T.C., Ropers H.H., Nuber U.A. (2003). Comparative study of methyl-CpG-binding domain proteins. BMC Genom..

[11] Muñoz E.M., de Souza F.S.J., Rath M.F., Martínez Cerdeño V. (2022). Editorial: Transcription Regulation—Brain Development and Homeostasis—A Finely Tuned and Orchestrated Scenario in Physiology and Pathology. Front. Mol. Neurosci..

[12] Qin S., Min J. (2014). Structure and function of the nucleosome-binding PWWP domain. Trends Biochem. Sci..

[13] Seabra C.M., Aneichyk T., Erdin S., Tai D.J.C., De Esch C.E.F., Razaz P., An Y., Manavalan P., Ragavendran A., Stortchevoi A. (2020). Transcriptional consequences of MBD5 disruption in mouse brain and CRISPR-derived neurons. Mol. Autism.

[14] Frasca A., Spiombi E., Palmieri M., Albizzati E., Valente M.M., Bergo A., Leva B., Kilstrup-nielsen C., Bianchi F., Di Carlo V. (2020). MECP 2 mutations affect ciliogenesis: A novel perspective for Rett syndrome and related disorders. EMBO Mol. Med..

[15] Lee B., Panda S., Lee H.Y. (2020). Primary Ciliary Deficits in the Dentate Gyrus of Fragile X Syndrome. Stem Cell Rep..

[16] Tereshko L., Turrigiano G.G., Sengupta P. (2022). Primary cilia in the postnatal brain: Subcellular compartments for organizing neuromodulatory signaling. Curr. Opin. Neurobiol..

[17] Truong M.E., Bilekova S., Choksi S.P., Li W., Bugaj L.J., Xu K., Reiter J.F., Truong M.E., Bilekova S., Choksi S.P. (2021). Article Vertebrate cells differentially interpret ciliary and extraciliary cAMP ll ll Article Vertebrate cells differentially interpret ciliary and extraciliary cAMP. Cell.

[18] Sheu S.-H., Upadhyayula S., Dupuy V., Pang S., Deng F., Wan J., Walpita D., Pasolli H.A., Houser J., Sanchez-Martinez S. (2022). A serotonergic axon-cilium synapse drives nuclear signaling to alter chromatin accessibility. Cell.

[19] Guo J., Otis J.M., Higginbotham H., Monckton C., Cheng J.G., Asokan A., Mykytyn K., Caspary T., Stuber G.D., Anton E.S. (2017). Primary Cilia Signaling Shapes the Development of Interneuronal Connectivity. Dev. Cell.

[20] Valente E.M., Rosti R.O., Gibbs E., Gleeson J.G. (2013). Primary cilia in neurodevelopmental disorders. Nat. Rev. Neurol..

[21] Youn Y.H., Han Y.G. (2018). Primary Cilia in Brain Development and Diseases. Am. J. Pathol..

[22] Hasenpusch-Theil K., Theil T. (2021). The Multifaceted Roles of Primary Cilia in the Development of the Cerebral Cortex. Front. Cell Dev. Biol..

[23] Andreu-Cervera A., Catala M., Schneider-Maunoury S. (2021). Cilia, ciliopathies and hedgehog-related forebrain developmental disorders. Neurobiol. Dis..

[24] Olstad E.W., Ringers C., Hansen J.N., Wens A., Brandt C., Wachten D., Yaksi E., Jurisch-Yaksi N. (2019). Ciliary Beating Compartmentalizes Cerebrospinal Fluid Flow in the Brain and Regulates Ventricular Development. Curr. Biol..

[25] Higginbotham H., Guo J., Yokota Y., Umberger N.L., Su C.Y., Li J., Verma N., Hirt J., Ghukasyan V., Caspary T. (2013). Arl13b-regulated cilia activities are essential for polarized radial glial scaffold formation. Nat. Neurosci..

[26] Baudoin J.P., Viou L., Launay P.S., Luccardini C., Espeso Gil S., Kiyasova V., Irinopoulou T., Alvarez C., Rio J.P., Boudier T. (2012). Tangentially Migrating Neurons Assemble a Primary Cilium that Promotes Their Reorientation to the Cortical Plate. Neuron.

[27] Guo J., Otis J.M., Suciu S.K., Stuber G.D., Caspary T., Anton E.S., Guo J., Otis J.M., Suciu S.K., Catalano C. (2019). Primary Cilia Signaling Promotes Axonal Tract Development and Is Disrupted in Joubert Syndrome- Article Primary Cilia Signaling Promotes Axonal Tract Development and Is Disrupted in Joubert Syndrome-Related Disorders Models. Dev. Cell.

[28] Lancaster M.A., Gleeson J.G. (2009). The primary cilium as a cellular signaling center: Lessons from disease. Curr. Opin. Genet. Dev..

[29] Louvi A., Grove E.A. (2011). Cilia in the CNS: The quiet organelle claims center stage. Neuron.

[30] Guo J., Higginbotham H., Li J., Nichols J., Hirt J., Ghukasyan V., Anton E.S. (2015). Developmental disruptions underlying brain abnormalities in ciliopathies. Nat. Commun..

[31] Shi M., Zhang H., Wang L., Zhu C., Sheng K., Du Y., Wang K., Dias A., Chen S., Whitman M. (2015). Premature termination codons are recognized in the nucleus in a reading-frame-dependent manner. Cell Discov..

[32] Han X., Wei Y., Wang H., Wang F., Ju Z., Li T. (2018). Nonsense-mediated mRNA decay: A “nonsense” pathway makes sense in stem cell biology. Nucleic Acids Res..

[33] Howden S.E., Maufort J.P., Duffin B.M., Elefanty A.G., Stanley E.G., Thomson J.A. (2015). Simultaneous Reprogramming and Gene Correction of Patient Fibroblasts. Stem Cell Rep..

[34] Zhang X., Li Z., Liu Y., Gai Z. (2020). Great expectations: Induced pluripotent stem cell technologies in neurodevelopmental impairments. Int. J. Med. Sci..

[35] Taapken S.M., Nisler B.S., Newton M.A., Sampsell-Barron T.L., Leonhard K.A., McIntire E.M., Montgomery K.D. (2011). Karotypic abnormalities in human induced pluripotent stem cells and embryonic stem cells. Nat. Biotechnol..

[36] Walker T., Huang J., Young K. (2016). Neural Stem and Progenitor Cells in Nervous System Function and Therapy. Stem Cells Int..

[37] Park S.M., Lim J.S., Ramakrishina S., Kim S.H., Kim W.K., Lee J., Kang H.C., Reiter J.F., Kim D.S., Kim H. (2018). Brain Somatic Mutations in MTOR Disrupt Neuronal Ciliogenesis, Leading to Focal Cortical Dyslamination. Neuron.

[38] Kossoff E.H., Thiele E.A., Pfeifer H.H., McGrogan J.R., Freeman J.M. (2005). Tuberous Sclerosis Complex and the Ketogenic Diet. Epilepsia.

[39] Coppola G., Klepper J., Ammendola E., Fiorillo M., della Corte R., Capano G., Pascotto A. (2006). The effects of the ketogenic diet in refractory partial seizures with reference to tuberous sclerosis. Eur. J. Paediatr. Neurol..

[40] Grocott O.R., Herrington K.S., Pfeifer H.H., Thiele E.A., Thibert R.L. (2017). Low glycemic index treatment for seizure control in Angelman syndrome: A case series from the Center for Dietary Therapy of Epilepsy at the Massachusetts General Hospital. Epilepsy Behav..

[41] Liebhaber G.M., Riemann E., Matthias Baumeister F.A. (2003). Ketogenic Diet in Rett Syndrome. J. Child Neurol..

[42] Rho J.M. (2017). How does the ketogenic diet induce anti-seizure effects?. Neurosci. Lett..

[43] Olson C.A., Vuong H.E., Yano J.M., Liang Q.Y., Nusbaum D.J., Hsiao E.Y. (2018). The Gut Microbiota Mediates the Anti-Seizure Effects of the Ketogenic Diet. Cell.

[44] Zhang Y., Zhou S., Zhou Y., Yu L., Zhang L., Wang Y. (2018). Altered gut microbiome composition in children with refractory epilepsy after ketogenic diet. Epilepsy Res..

[45] Calderón N., Betancourt L., Hernández L., Rada P. (2017). A ketogenic diet modifies glutamate, gamma-aminobutyric acid and agmatine levels in the hippocampus of rats: A microdialysis study. Neurosci. Lett..

[46] Bough K.J., Wetherington J., Hassel B., Pare J.F., Gawryluk J.W., Greene J.G., Shaw R., Smith Y., Geiger J.D., Dingledine R.J. (2006). Mitochondrial biogenesis in the anticonvulsant mechanism of the ketogenic diet. Ann. Neurol..

[47] Chen C.X.-Q., Abdian N., Maussion G., Thomas R.A., Demirova I., Cai E., Tabatabaei M., Beitel L.K., Karamchandani J., Fon E.A. (2021). A Multistep Workflow to Evaluate Newly Generated iPSCs and Their Ability to Generate Different Cell Types. Methods Protoc..

[48] Namba T., Huttner W.B. (2017). Neural progenitor cells and their role in the development and evolutionary expansion of the neocortex. Wiley Interdiscip. Rev. Dev. Biol..

[49] Martínez-Cerdeño V., Noctor S.C. (2018). Neural progenitor cell terminology. Front. Neuroanat..

[50] Suzuki H., Imajo Y., Funaba M., Nishida N., Sakamoto T., Sakai T. (2022). Current Concepts of Neural Stem/Progenitor Cell Therapy for Chronic Spinal Cord Injury. Front. Cell. Neurosci..

[51] Mullegama S.V., Klein S.D., Williams S.R., Innis J.W., Probst F.J., Haldeman-Englert C., Martinez-Agosto J.A., Yang Y., Tian Y., Elsea S.H. (2021). Transcriptome analysis of MBD5-associated neurodevelopmental disorder (MAND) neural progenitor cells reveals dysregulation of autism-associated genes. Sci. Rep..

[52] Tang F., Zhang X., Ke P., Liu J., Zhang Z., Hu D., Gu J., Zhang H., Guo H., Zang Q. (2023). MBD5 regulates NMDA receptor expression and seizures by inhibiting Stat1 transcription. Neurobiol. Dis..

[53] Shiwaku H., Umino A., Umino M., Nishikawa T. (2017). Phencyclidine-induced dysregulation of primary cilia in the rodent brain. Brain Res..

[54] Varga E., Nemes C., Bock I., Táncos Z., Berzsenyi S., Lévay G., Román V., Kobolák J., Dinnyés A. (2017). Establishment of an induced pluripotent stem cell ( iPSC ) line from a 9-year old male with autism spectrum disorder (ASD). Stem Cell Res..

[55] Caspary T., Larkins C.E., Anderson K.V. (2007). The Graded Response to Sonic Hedgehog Depends on Cilia Architecture. Dev. Cell.

[56] Mishra P., Singh U., Pandey C., Mishra P., Pandey G. (2019). Application of student’s t-test, analysis of variance, and covariance. Ann. Card. Anaesth..

[57] Almuzzaini B., Alatwi N.S., Alsaif S., Al Balwi M.A. (2020). A novel interstitial deletion of chromosome 2q21.1-q23.3: Case report and literature review. Mol. Genet. Genom. Med..

[58] Bonnet C., Ali Khan A., Bresso E., Vigouroux C., Béri M., Lejczak S., Deemer B., Andrieux J., Philippe C., Moncla A. (2013). Extended spectrum of MBD5 mutations in neurodevelopmental disorders. Eur. J. Hum. Genet..

[59] Bravo-Oro A., Lurie I.W., Elizondo-Cárdenas G., Peña-Zepeda C., Salazar-Martínez A., Correa-González C., Castrillo J.L., Avila S., Esmer C. (2015). A novel interstitial deletion of 2q22.3 q23.3 in a patient with dysmorphic features, epilepsy, aganglionosis, pure red cell aplasia, and skeletal malformations. Am. J. Med. Genet. A.

[60] Castro-Gago M., Gómez-Lado C., Barros-Angueira F., Trujillo-Ariza M.V., Fuentes-Pita P., López-Vázquez A.M., Eirís-Puñal J. (2019). De novo heterozygous mutation in the MBD5 gene associated with bilateral band heterotopia and polymicrogyria. Rev. Neurol..

[61] Chung B.H.Y., Stavropoulos J., Marshall C.R., Weksberg R., Scherer S.W., Yoon G. (2011). 2q23 de novo microdeletion involving the MBD5 gene in a patient with developmental delay, postnatal microcephaly and distinct facial features. Am. J. Med. Genet. Part A.

[62] Chung B.H.Y., Mullegama S., Marshall C.R., Lionel A.C., Weksberg R., Dupuis L., Brick L., Li C., Scherer S.W., Aradhya S. (2012). Severe intellectual disability and autistic features associated with microduplication 2q23.1. Eur. J. Hum. Genet..

[63] Cukier H.N., Lee J.M., Ma D., Young J.I., Mayo V., Butler B.L., Ramsook S.S., Rantus J.A., Abrams A.J., Whitehead P.L. (2012). The Expanding Role of MBD Genes in Autism: Identification of a MECP2 Duplication and Novel Alterations in MBD5, MBD6, and SETDB1. Autism Res..

[64] De Vries B.B.A., Pfundt R., Leisink M., Koolen D.A., Vissers L.E.L.M., Janssen I.M., Van Reijmersdal S., Nillesen W.M., Huys E.H.L.P.G., De Leeuw N. (2005). Diagnostic genome profiling in mental retardation. Am. J. Hum. Genet..

[65] Du X., An Y., Yu L., Liu R., Qin Y., Guo X., Sun D., Zhou S., Wu B., Jiang Y. (2014). A genomic copy number variant analysis implicates the MBD5 and HNRNPU genes in Chinese children with infantile spasms and expands the clinical spectrum of 2q23.1 deletion. BMC Med. Genet..

[66] Fry A.E., Rees E., Thompson R., Mantripragada K., Blake P., Jones G., Morgan S., Jose S., Mugalaasi H., Archer H. (2016). Pathogenic copy number variants and SCN1A mutations in patients with intellectual disability and childhood-onset epilepsy. BMC Med. Genet..

[67] Gokben S., Onay H., Yilmaz S., Atik T., Serdaroglu G., Tekin H., Ozkinay F. (2017). Targeted next generation sequencing: The diagnostic value in early-onset epileptic encephalopathy. Acta Neurol. Belg..

[68] Hamdan F.F., Srour M., Capo-Chichi J.-M., Daoud H., Nassif C., Patry L., Massicotte C., Ambalavanan A., Spiegelman D., Diallo O. (2014). De novo mutations in moderate or severe intellectual disability. PLoS Genet..

[69] Han J.Y., Lee I.G., Jang W., Kim M., Kim Y., Jang J.H., Park J. (2017). Diagnostic exome sequencing identifies a heterozygous MBD5 frameshift mutation in a family with intellectual disability and epilepsy. Eur. J. Med. Genet..

[70] Ishizuka K., Kimura H., Yoshimi A., Banno M., Kushima I., Uno Y., Okada T., Mori D., Aleksic B., Ozaki N. (2016). Investigation of single-nucleotide variants in MBD5 associated with autism spectrum disorders and schizophrenia phenotypes. Nagoya J. Med. Sci..

[71] Jaillard S., Dubourg C., Gérard-Blanluet M., Delahaye A., Pasquier L., Dupont C., Henry C., Tabet A.C., Lucas J., Aboura A. (2009). 2q23.1 microdeletion identified by array comparative genomic hybridisation: An emerging phenotype with Angelman-like features?. J. Med. Genet..

[72] Kushima I., Aleksic B., Nakatochi M., Shimamura T., Shiino T., Yoshimi A., Kimura H., Takasaki Y., Wang C., Xing J. (2017). High-resolution copy number variation analysis of schizophrenia in Japan. Mol. Psychiatry.

[73] Le T.N.U., Ha T.M.T. (2021). MBD5-related intellectual disability in a Vietnamese child. Am. J. Med. Genet. Part A.

[74] Motobayashi M., Nishimura-Tadaki A., Inaba Y., Kosho T., Miyatake S., Niimi T., Nishimura T., Wakui K., Fukushima Y., Matsumoto N. (2012). Neurodevelopmental features in 2q23.1 microdeletion syndrome: Report of a new patient with intractable seizures and review of literature. Am. J. Med. Genet. Part A.

[75] Mullegama S.V., Rosenfeld J.A., Orellana C., Van Bon B.W.M.M., Halbach S., Repnikova E.A., Brick L., Li C., Dupuis L., Rosello M. (2014). Reciprocal deletion and duplication at 2q23.1 indicates a role for MBD5 in autism spectrum disorder. Eur. J. Hum. Genet..

[76] Mullegama S.V., Elsea S.H. (2014). Intragenic MBD5 familial deletion variant does not negatively impact MBD5 mRNA expression. Mol. Cytogenet..

[77] Myers K.A., Marini C., Carvill G.L., McTague A., Panetta J., Stutterd C., Stanley T., Marin S., Nguyen J., Barba C. (2021). Phenotypic spectrum of seizure disorders in MBD5-associated neurodevelopmental disorder. Neurol. Genet..

[78] Noh G.J., Graham J.M.J. (2012). 2q23.1 microdeletion of the MBD5 gene in a female with seizures, developmental delay and distinct dysmorphic features. Eur. J. Med. Genet..

[79] Ohori S., Tsuburaya R.S., Kinoshita M., Miyagi E., Mizuguchi T., Mitsuhashi S., Frith M.C., Matsumoto N. (2021). Long-read whole-genome sequencing identified a partial MBD5 deletion in an exome-negative patient with neurodevelopmental disorder. J. Hum. Genet..

[80] Orrico A., Galli L., Rossi M., Cortesi A., Mazzi M., Caterino E. (2021). The Variable Expression of a Novel MBD5 Gene Frameshift Mutation in an Italian Family. Neuropediatrics.

[81] Shichiji M., Ito Y., Shimojima K., Nakamu H., Oguni H., Osawa M., Yamamoto T. (2013). A cryptic microdeletion including MBD5 occurring within the breakpoint of a reciprocal translocation between chromosomes 2 and 5 in a patient with developmental delay and obesity. Am. J. Med. Genet. Part A.

[82] Tadros S., Wang R., Waters J.J., Waterman C., Collins A.L., Collinson M.N., Ahn J.W., Josifova D., Chetan R., Kumar A. (2017). Inherited 2q23.1 microdeletions involving the MBD5 locus. Mol. Genet. Genom. Med..

[83] Turner T.N., Hormozdiari F., Duyzend M.H., McClymont S.A., Hook P.W., Iossifov I., Raja A., Baker C., Hoekzema K., Stessman H.A. (2016). Genome Sequencing of Autism-Affected Families Reveals Disruption of Putative Noncoding Regulatory DNA. Am. J. Hum. Genet..

[84] Van Bon B.W.M., Koolen D.A., Brueton L., McMullan D., Lichtenbelt K.D., Adès L.C., Peters G., Gibson K., Novara F., Pramparo T. (2010). The 2q23.1 microdeletion syndrome: Clinical and behavioural phenotype. Eur. J. Hum. Genet..

[85] Verhoeven W., Egger J., Kipp J., Verheul-Aan de Wiel J., Ockeloen C., Kleefstra T., Pfundt R. (2019). A novel MBD5 mutation in an intellectually disabled adult female patient with epilepsy: Suggestive of early onset dementia?. Mol. Genet. Genom. Med..

[86] Wagenstaller J., Spranger S., Lorenz-Depiereux B., Kazmierczak B., Nathrath M., Wahl D., Heye B., Glaser D., Liebscher V., Meitinger T. (2007). Copy-number variations measured by single-nucleotide-polymorphism oligonucleotide arrays in patients with mental retardation. Am. J. Hum. Genet..

[87] Wang Y., Du X., Bin R., Yu S., Xia Z., Zheng G., Zhong J., Zhang Y., Jiang Y.-H., Wang Y. (2017). Genetic Variants Identified from Epilepsy of Unknown Etiology in Chinese Children by Targeted Exome Sequencing. Sci. Rep..

[88] Williams S.R., Mullegama S.V., Rosenfeld J.A., Dagli A.I., Hatchwell E., Allen W.P., Williams C.A., Elsea S.H. (2010). Haploinsufficiency of MBD5 associated with a syndrome involving microcephaly, intellectual disabilities, severe speech impairment, and seizures. Eur. J. Hum. Genet..

[89] Woodbury-Smith M., Nicolson R., Zarrei M., Yuen R.K.C., Walker S., Howe J., Uddin M., Hoang N., Buchanan J.A., Chrysler C. (2017). Variable phenotype expression in a family segregating microdeletions of the NRXN1 and MBD5 autism spectrum disorder susceptibility genes. NPJ Genom. Med..

