# Supplementary material for: A Novel Genetic Variant in MBD5 Associated with Severe Epilepsy and Intellectual Disability: Potential Implications on Neural Primary Cilia
Source: Int J Mol Sci. 2023 Aug 9;24(16):12603. doi: 10.3390/ijms241612603 (PMC10454663; doi:10.3390/ijms241612603)
Supplement: Supplementary file 1 [file ijms-24-12603-s001.zip › ijms-2533004-supplementary.pdf]

Supplementary Material

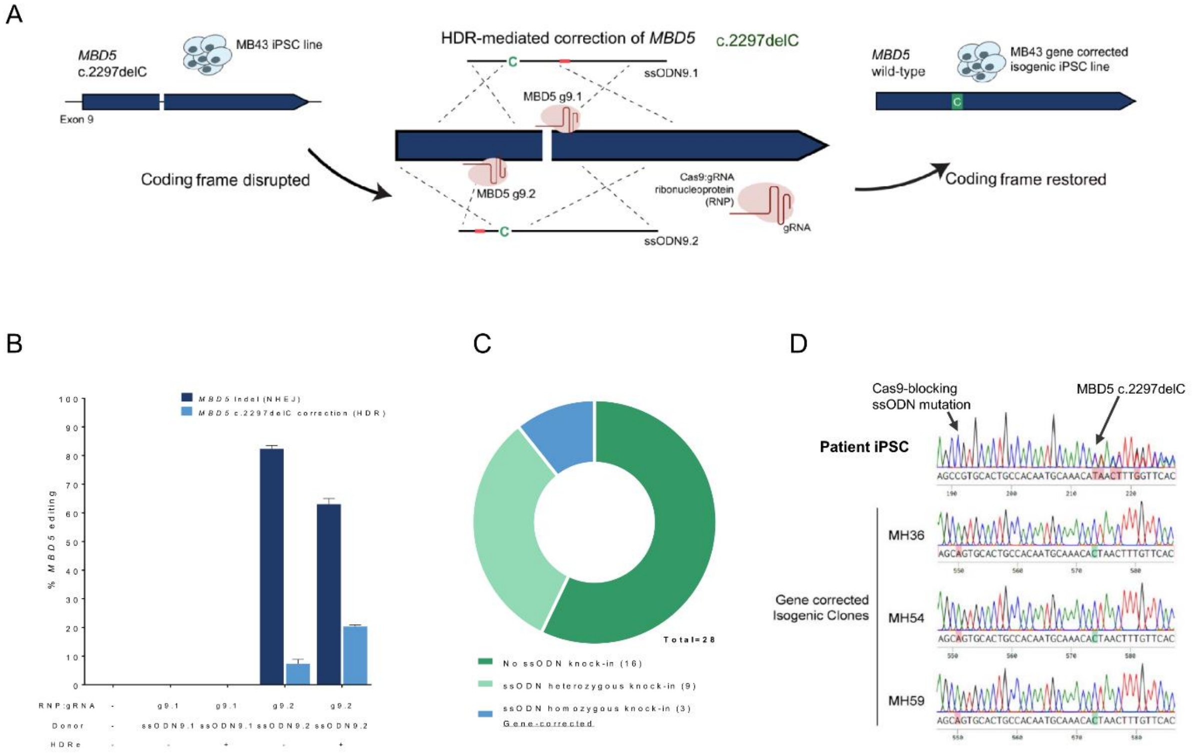

Figure S1 – Correction of *MBD5* c.2297delC in patient-derived induced pluripotent stem cells by CRISPR/Cas9-mediated homology-directed repair (HDR). A) Schematic illustration of the HDR-mediated correction of *MBD5* c.2297delC. B) Estimation of *MBD5* editing by Sanger sequence analysis of genomic DNA from patient-derived iPSCs treated with *MBD5*-targeting RNP and respective ssODN template, in the presence or absence of small-molecule HDR enhancer (HDR). Data represented as mean ± SD (N=2). C) Genotype of *MBD5* editing in iPS cell clones. Single cell-derived clones from the MBD9.2/ssODN9.2 treated population were isolated and genotyped by Sanger sequencing. D) Sequence alignment of *MBD5* gene-corrected isogenic iPS cell clones (MH36, MH54 and MH59) against parental patient-derived iPS cell line. Sequence position of c.2297delC and ssODN Cas9-blocking mutations are illustrated. Red boxes highlight variations against wild-type *MBD5*, while green boxes highlight the correction of c.2297delC in isogenic iPS cell lines.

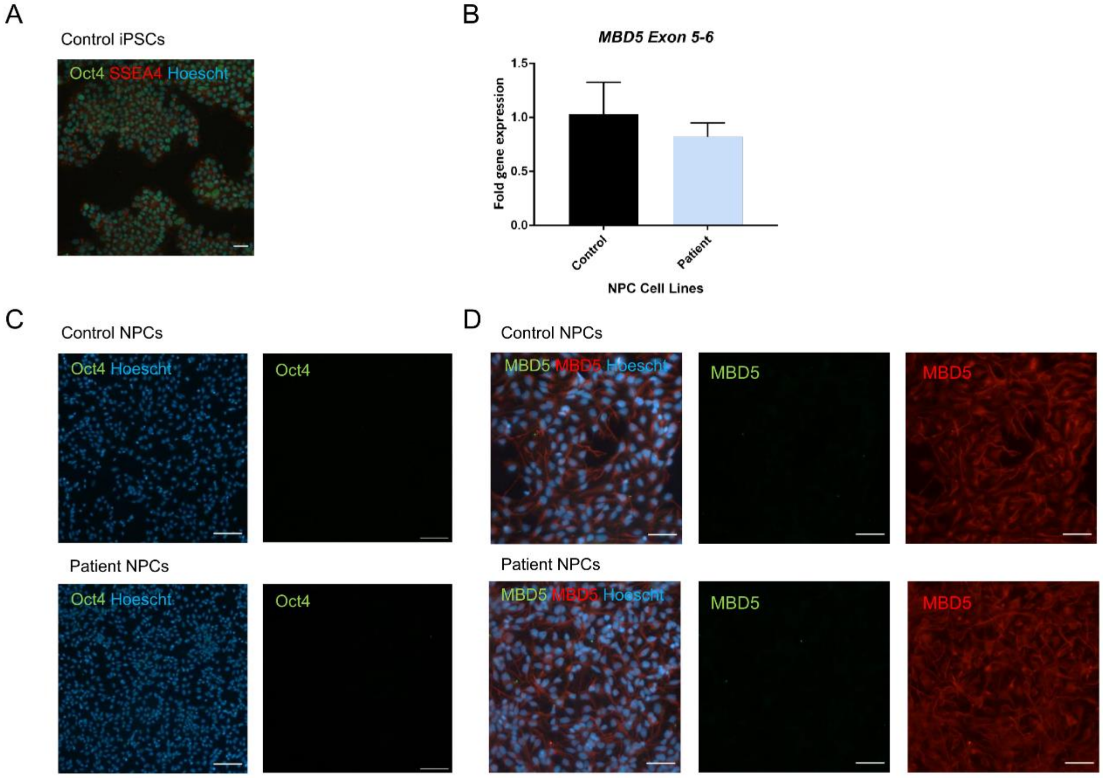

Figure S2 - A) CRISPR-Cas9-edited iPS cells maintain expression of pluripotency markers OCT4 and SSEA4 (objective: 20x, scale bar: 50 µm); B) *MBD5* expression profile of patient-derived and control NPCs, upstream of the deletion site; C) NPCs reveal absence of pluripotency marker OCT4 (objective: 20x, scale bar: 100 µm); D) NPCs do not reveal specific MBD5 staining (green: anti-MBD5 Proteintech, 15961-1-AP; red: anti-MBD5 Sigma-Aldrich WH0055777M1; scale bar: 100 µm).

Table S1 - ***MBD5*** **variant-associated phenotypes reported in the literature.**

MBD5 Variant	Sex	Reported Phenotype							Reference
		Developmental Delay	Intellectual Disability	Seizures	Autistic-like behaviors	Dysmorphic features	Behavior Problems	Hypotonia	
**Interstitial deletion at 2q21.1-2q23.3**	M			X		X			[57]
**Interstitial deletion at 2q23.1**	F	X	X		X	X	X		[58]
	F	X	X		X	X	X		
**Interstitial duplication at 2q23.1**	M	X	X		X	X			
**Interstitial deletion at 2q23.1**	F	X	X		X	X	X		
**Nucleotide substitution (c.440C>G)**	M	X	X	X	X	X		X	
**Interstitial Deletion at 2q22.3-2q23.3**	M	X		X		X		X	[59]
**Nucleotide substitution (c.397+1G>C)**	F	X		X				X	[60]
**Interstitial deletion at 2q23.1–23.3**	F	X		X		X	X		[61]
**Duplication at 2q23.1–2q23.2**	F	X	X		X	X	X	X	[62]
**Duplication at 2q22.3q23.2**	M	X	X		X	X		X	

MBD5 Variant	Sex	Reported Phenotype							Reference
		Developmental Delay	Intellectual Disability	Seizures	Autistic-like behaviors	Dysmorphic features	Behavior Problems	Hypotonia	
**Nucleotide substitution (c.1327G>A)**	M				X				[63]
**Nucleotide substitution (c.3740T>C)**	F	X			X				
**Nucleotide substitution (c.3806A>G)**	F	X	X	X	X		X		
	M	X	X		X		X		
	M	X			X		X		
**Nucleotide substitution (c.3896G>A)**	M				X				
**Deletion at 2q23.1q23.2**	F	X		X		X	X		[64]
**Deletion at 2q23.1**	F	X		X	X	X	X	X	[65]
**Deletion at 2q23.1**	F		X	X		X			[66]
**Nucleotide substitution (c.3595G>A)**	F	X		X	X	X			[67]
**Deletion in *MBD5* (c.340_347del.)**	M	X	X	X				X	[68]
**Frameshift variant (c.254_255delGA)**	F	X	X	X	X	X	X		[69]
	M			X					

***MBD5* Variant**	**Sex**	**Reported Phenotype**							**Reference**
		**Developmental Delay**	**Intellectual Disability**	**Seizures**	**Autistic-like behaviors**	**Dysmorphic features**	**Behavior Problems**	**Hypotonia**	
**Nucleotide substitution (c.-443G>A)**	M				X		X		[70]
**Nucleotide substitution (c.1862A>T)**	F				X				
**Nucleotide substitution (c.2550A>G)**	M			X					
**Nucleotide substitution (c.4045G>A)**	M	X					X		
**Interstitial deletion at 2q23.1**	M	X		X		X	X	X	[71]
**Interstitial deletion at 2q22.3-2q23.1**	M	X		X	X	X	X		
***MBD5* deletion**	M	X	X	X	X		X		[72]
	F	X	X	X	X				
	M	X	X	X	X				
	M	X	X	X	X				
	F	X	X	X	X				
**Nucleotide substitution (c.217-1G>C)**	M	X	X	X	X				[73]
**Deletion at 2q23.1–2q23.2**	M	X	X	X		X			[74]

MBD5 Variant	Sex	Reported Phenotype							Reference
		Developmental Delay	Intellectual Disability	Seizures	Autistic-like behaviors	Dysmorphic features	Behavior Problems	Hypotonia	
**Duplication at 2q23.1**	F	X		X	X	X	X	X	[75]
**Duplication at 2q23.1**	M	X		X	X	X	X	X	
**Duplication including 2q22.3- 2q23.3**	F	X		X	X	X	X	X	
**Duplication including 2q22.3- 2q23.2**	M	X			X	X	X	X	
**Duplication including 2q22.3- 2q23.1**	M	X			X	X		X	
**Deletion at 2q23.1**	M	X		X	X	X	X	X	[76]
**Complete deletion of *MBD5***	M	X	X	X			X		[77]
**Proximal deletion of *MBD5***	M	X	X	X					
**Distal deletion of *MBD5***	M	X	X	X			X		
**Proximal deletion of *MBD5***	F	X	X	X			X		
**p.Thr157Glnfs*4 truncation**	F	X	X	X			X		
**Proximal deletion of *MBD5***	F	X	X	X			X		

MBD5 Variant	Sex	Reported Phenotype							Reference
		Developmental Delay	Intellectual Disability	Seizures	Autistic-like behaviors	Dysmorphic features	Behavior Problems	Hypotonia	
**Proximal deletion of *MBD5***	M	X	X	X			X		
**Proximal deletion of *MBD5***	M	X	X	X			X		
**Complete deletion of *MBD5***	M	X	X	X					
**Proximal deletion of *MBD5***	F	X	X	X					
**Complete deletion of *MBD5***	F	X	X	X			X		
**Proximal deletion of *MBD5***	M	X	X	X					
**Proximal deletion of *MBD5***	F	X	X	X			X		
**Proximal deletion of *MBD5***	M	X	X	X			X		
**Proximal deletion of *MBD5***	M	X	X	X			X		
**Proximal deletion of *MBD5***	M	X	X	X			X		
**Distal duplication**	F	X		X					
**Distal deletion of *MBD5***	M	X	X	X			X		

***MBD5* Variant**	**Sex**	**Reported Phenotype**							**Reference**
		**Developmental Delay**	**Intellectual Disability**	**Seizures**	**Autistic-like behaviors**	**Dysmorphic features**	**Behavior Problems**	**Hypotonia**	
**Nucleotide substitution (c.598C>T)**	M	X	X	X			X		
**Proximal deletion of *MBD5***	M	X	X	X			X		
**Complete deletion of *MBD5***	M	X	X	X					
**Intragenic duplication**	F	X	X	X					
**Distal deletion of *MBD5***	M	X	X	X			X		
**Deletion at 2q23.1**	F	X		X		X		X	[78]
**Deletion of *MBD5***	M	X	X	X	X	X			[79]
**Deletion leading to a frameshift in *MBD5* (c.2579del.)**	M	X	X		X	X			[80]
	M	X	X	X		X			
	F		X						
**Reciprocal translocation between chromosomes 2 and 5, deletion at *MBD5***	F	X		X	X	X	X	X	[81]
**Deletion at 2q23.1**	F	X		X					[82]
**Deletion at 2q23.1**	M	X		X		X			

MBD5 Variant	Sex	Reported Phenotype							Reference
		**Developmental Delay**	**Intellectual Disability**	**Seizures**	**Autistic-like behaviors**	**Dysmorphic features**	**Behavior Problems**	**Hypotonia**	
	F	X				X			
	F	X				X			
**Deletion at 2q23.1**	M		X	X	X				
**Deletion at q22.3-q23.1**	M	X							
***MBD5*-specific disruptions and 2q23.1 deletions**	Cohort of 65 M and F	X		X	X	X	X	X	[6]
**Intronic deletion of MBD5**					X				[83]
**Deletions at 2q23.1**	F	X		X	X	X			[84]
	F	X		X	X	X			
	F	X			X	X			
	M	X		X	X	X	X	X	
	F	X		X	X	X	X		
	M	X		X	X	X			
	M	X		X	X	X			
	M	X			X	X	X	X	

MBD5 Variant	Sex	Reported Phenotype							Reference
		Developmental Delay	Intellectual Disability	Seizures	Autistic-like behaviors	Dysmorphic features	Behavior Problems	Hypotonia	
	F	X		X	X	X		X	
	F	X		X	X	X	X		
	M	X		X					
**Frameshift variant (c.2173del.)**	F	X	X	X	X		X		[85]
***MBD5* deletion**	M	X		X					[86]
**Nucleotide substitution (c.365C>T)**	*F*		*X*	*X*		*X*			[87]
**Nucleotide substitution (c.1885A>G)**	M	X		X					
**Deletion at 2q23.1**	F	X	X	X		X	X	X	[88]
**Deletion of chromosome 2q22.3–q23.3**	F	X	X	X		X	X	X	
***MBD5* deletion**	M		X		X				[89]

Table S2 - Primers used for Sanger Sequencing

**Name**	**Sequence 5' - 3'**	**Tm (°C)**
***MBD5* Ex9-F**	TGCTAAGGAAGCAGGGTCAGG	57.99
***MBD5* Ex9-F2**	TCGAGCAGCACTAAGAGATAAGC	59.17
***MBD5* Ex9-R**	ATCTTCCCAATAGACAAATCCCTAGC	56.15
***MBD5* Ex9-R2**	ACAAACCAGGAAACCCTGCC	57.09

Table S3 - *MBD5*-targeting guide RNA (gRNA) and single-stranded oligodeoxynucleotide (ssODN) sequences used for MBD5 c.2297delC correction.

**gRNAs and ssODNs**	**Sequences 5' - 3'**
***MBD5* g9.1**	ACAAAGTTATGTTTGCATTG
***MBD5* ssODN9.1**	CTGCTAACCAGCTGCATTTTACAGATCCCAGTATGAACTCTAGT GTTCTTCAGAACATACCTTTAAGAGGGGAAGCCGTGCACTGTC ACAATGCAAACACTAACTTTGTTCACAGTAACAGTCCAGT
***MBD5* g9.2**	GTTTGCATTGTGGCAGTGCA
***MBD5* ssODN9.2**	TTGCCTTGCTCTGCTAACCAGCTGCATTTTACAGATCCCAGTAT GAACTCTAGTGTTCTTCAGAACATACCTTTAAGAGGGGAAGCA GTGCACTGCCACAATGCAAACACTAACTTTGTTCACAGTA

Table S4 - Primers used for genotyping *MBD5* c.2297Cdel correction.

**Primers**	**Sequences 5' - 3'**	**Tm (°C)**
***MBD5* Ex9-F3**	TTGAACCAGCACAATGCTGC	59.97
***MBD5* Ex9-R**	ATCTTCCCAATAGACAAATCCCTAGC	60.47

Table S5 - Primers used for *MBD5* gene expression assessment.

Name	FW Sequence 5' - 3'	RV Sequence 5' - 3'	Product Size (bps)
MBD5 - Exon 12	TTCAGAACTTTCAGGTGAGAATGC	TTGCAACTCACAAGGTGTATCTTG	140
GAPDH	CATGAGAAGTATGACAACAGCCT	AGTCCTTCCACGATACCAAAGT	113

Table S6 – NGS panel of sequenced genes.

**Genes assessed via array panel**
AARS, ACY1, AKT3, ALDH7A1, ALG13, AMT, AP3B2, ARFGEF2, ARMGEF15, ARHGEF9, ARV1, ARX, ATPIA2, ATPGAP2, AUH, BCKDK, BRAT1, BSCL2, CACNA1A, CACNA1, CACNA2D2, CACNB4, CAD, CDK5, CDKL5, CHD2, CHRNA2, CHRNA4, CHRNA7, CHRNB2, CLCN2, CLCN4, CLN3, CLN5, CLN6, CLN8, CNTNAP2, COL4Al, COL4A2, COX15, CPA6, CPT2, CRADD, CSF1R, CSTB, CTSD, CTSF, DARS2, DCX, DENND5A, DEPDC5, DNAJC5, DNM1, DNM1L, DOCK7, DYNC1H1, DYRK1A, EEF1A2, EFHC1, EIF2Bl, EIF2B2, EIF2B3, EIF2B4, EIF2B5, EMX2, EPM2A, ERBB4, ETHE1, FADD, FARS2, FBXL4, FGF12, FGFR1, FLNA, FOLRl, FOXGJ, FRRS1L, GABRAl, GABRBl, GABRB2, GABRB3, GABRD, GABRG2, GAMT, GATM, GCSH, GLDC, GLI2, GNAOl, GOSR2, GRIN1, GRIN2A, GRIN2B, GRIN2D, GUFl, HCNl, HCN2, HEPACAM, HNRNPU, HTR2A, HTRA1, IQSEC2, IRF3, ITPA, KANSL1, KARS, KATNBl, KCNA2, KCNB1, KCNH2, KCNH5, KCNJIO, KCNQ2, KCNQ3, KCNTl, KCNT2, KCTD7, KPNA7, LAMBl, LGI3, LIAS, LIPT2, MAGI2, MBD5. MDH2, MECP2. MFF2C, MFF, MFSD8, MLC1, MTOR, NAXE, NDEl, NDP, NECAPl, NEDD4L, NHLRC1, NOTCH3, NR2F1, NRXNl, NTRK2, PAFAH1B1, PCDH19, PIGA, PIGP, PIGQ, PIGV, PIK3CA, PIK3R2, PLCBl, PNKP, PNPO, POLG, PPP3CA, PPT1, PRICKLE1, PRRT2, PTCHl, PTEN, QARS, RANBP2, RBFOXl, RBFOX3, RELN, RNASET2, ROGDI, RRM2B, SCARB2, SCNlA, SCNlB, SCN2A, SCN3A, SCN5A, SCN8A, SCN9A, SCO2, SERACl, SERPINI1, SHH, SIKl, SIX3, SLC12A5, SLC13A5, SLC19A3, SLC1A2, SLC25A12, SLC25A22, SLC2A1, SLC35A2, SLC6Al, SLC6A8, SlC6A9, SLC9A6, SPTAN1, SRPX2, ST3GAL3, ST3GAL5, STRADA, STXlB, STXBPl, SUCLA2, SYNl, SYNGAPl, SYNE1, SZT2, TANGO2, TBC1D24, TBC1D7, TBCD, TBCE, TCF4, TMTC3, TNK2, TPK1, TPP1, TRAF3, TRAPPC12, TSC1, TSC2, TUBAlA, UBA5, UBE3A, WDR45, WWOX, YWHAG, ZEB2
